# Effects of Mindfulness for Performance Programme on Actual Performance in Ecological Sport Context: Two Studies in Basketball and Table Tennis

**DOI:** 10.3390/ijerph191912950

**Published:** 2022-10-10

**Authors:** Karima Tebourski, Marjorie Bernier, Manel Ben Salha, Nizar Souissi, Jean F. Fournier

**Affiliations:** 1 Activité Physique Sport et Santé, Observatoire National du Sport, 1003 Tunis, Tunisia; 2 Faculté des Sciences du Sport, CREAD, EA 3875, Université de Bretagne Occidentale, 29238 Brest, France; 3 UFR STAPS, LINP2, Université Paris Nanterre, 92001 Nanterre, France

**Keywords:** Mindfulness, performance, basketball, table tennis

## Abstract

Mindfulness For Performance is a programme that has been developed over 15 years. It aims to help athletes maintain effective attentional focus regardless of the disruptive sensations and thoughts induced by the performance situation. It is inspired by Mindfulness Based Stress Reduction and Acceptance Commitment Therapy programmes and has been adapted to the specificities of sport. It is composed of three steps: (a) psychoeducation and identification of the focus of attention, (b) mindfulness and acceptance training, and (c) integrating skills acquired into training and competition. This article reports the effects of MFP in two studies in national basketball players and in young table tennis players. The first study showed that mindfulness skills and free-throw accuracy during basketball games increased more in the experimental group than in the control group. Table tennis results revealed that participants who showed the highest percentage of adherence to the programme benefited more from MFP training in terms of performance outcome (i.e., accumulated points collected from published results compared with the baseline phase) than participants who showed weaker percentages of adherence to the programme. Both studies provided some evidence on the effects of MFP on specific performance indicators (i.e., free-throw accuracy in basketball and ranking points in table tennis), but this needs to be confirmed by further research measuring other relevant performance indicators. The impact and conditions of adherence also deserve more consideration.

## 1. Introduction

Mindfulness and Acceptance Based Interventions (MABIs) have proliferated in sport over the past few decades. The fundamental objectives of MABIs are to enhance the awareness of one’s experiences and to modify one’s relation to internal experiences [1]. They are considered to be a promising approach to developing psychological skills, as they meet the requirements of sport performance [2, 3, 4]. Using diverse protocols, it was shown that MABIs improve psychological performance-related variables (for reviews, see [3,5,6,7]), such as attention regulation [8,9], emotion regulation [10] and decreasing anxiety [11,12]. However, effects of MABIs on performance are not supported by strong empirical evidence. While several studies have demonstrated the positive effects of MABIs on subjective performances [10,13], the evidence of their benefits for objective performances is unclear. 

The effects of MABIs on performance were mainly examined in precision sports or tasks such as shooting [14, 15, 16], free throw [17,18], accuracy in tennis service [19], and golf putting [20]. Two studies assessed MABIs’ effects on endurance tasks in experimental conditions(rowing test [21], and graded exercise test [22]). The studies examining MABIs’ benefits on actual competition performance in real sport contexts remain very scarce. They concerned shooting [14], running [12], golf [8], figure skating [23] and swimming [9]. They showed inconsistent results and presented a high risk of bias [6]. Thus, empirical evidence needs to be underpinned. Considering the specific characteristics of real competition performance (high stakes, stress, uncertainty, complexity, variability), it is important to verify that MABIs significantly help athletes to improve performance. Thus, it is necessary to reinforce the evidence of MABIs effects in high ecological sport situations.

Among MABIs developed in sport (e.g., Mindfulness Acceptance Commitment, [24]; Mindfulness Sport Performance Enhancement, [25]; Mindfulness Meditation Training for Sport, [26]), the Mindfulness For Performance (MFP) programme is specifically designed to improve sport performance [27]. Unlike other MABIs for sport, the MFP programme has neither clinical focus nor is intended to improve wellbeing. The goal is to help athletes concentrate on efficient attentional foci in high-demanding situations, regardless of the cognitions, sensations, and emotions that may disturb them [27]. The MFP programme is based on the programme developed by Bernier et al. [8,23]. The intervention lasts 6 weeks and consists of three main stages: (a) identification of foci of attention, (b) mindfulness and acceptance training, and (c) integration into training and competition. The first stage aims to define and use relevant foci of attention. Athletes are helped to identify their relevant focus of attention according to the scientific evidence on the efficacy of the different kinds of focus on performance. Indeed, a strong body of literature suggests that an external focus is better than an internal one for performing automatized skills. However, several articles have suggested that the efficacy of external focus is not self-evident in a naturalistic sport context [28,29,30,31]. Thus, in this stage of the MFP programme, adopting an external focus is recommended, but the identification of the focus of attention is also individualized and adapted to the nature of the task and the automaticity level of the action. Athletes are, therefore, invited to identify the foci they usually use, then test their efficacy, and finally replace the inefficient or detrimental ones by other, more relevant ones. The more efficient foci are retained and used in different training situations to anchor them. During the stages that followed, their efficacy is checked regularly, and they can be adjusted as needed. The second stage of the MFP programme consists of training mindfulness and acceptance skills. Mindfulness learning and practice is introduced with very brief exercises (one-minute scans) and ten-minute meditation exercises each day. Then, acceptance practice is introduced to help athletes performing with distractions (unwanted thoughts or sensations) rather than avoiding or replacing them. It is trained by ten-minute daily exercises of acceptance using metaphors (train metaphor, cinema metaphor). Then, mindfulness and acceptance exercises get shorter and alternate between focusing on breathing and switching to a voluntary distraction (e.g., reading, checking phone). As in a competition context, the goal is to learn to refocus quickly on the task at hand when disturbances (e.g., thoughts related to emotions) occur. The third stage aims to integrate mindfulness and acceptance skills into practice and competition. Throughout the six weeks, the scans get shorter and more frequent, and are placed at appropriate moments of training sessions or competitions. During this stage, athletes train to apply mindfulness and acceptance strategies (e.g., scan to monitor their attention, metaphors to reduce reactivity and accept internal events) in high-demand situations (e.g., high-stake situations). Athletes are invited to adopt these strategies in competitions to keep their attention to the task at hand and refocus quickly on the foci defined in the first stage when they are disturbed by internal events (thoughts, emotions, sensations). Authors of the MFP programme recommend to adapt it to the specific requirements of the discipline and to integrate it as far as possible in sport training sessions [8,23,27]. 

Preliminary evidence of the efficacy of MFP was demonstrated in two studies that had set up original versions of the programme [8,23]. Bernier et al. [8] showed that young golfers who followed MFP significantly improved their activation regulation skills—measured by OMSAT-3* [32]—compared to a control group who followed a traditional psychological skills training. The golfers in the MFP group all enhanced their national ranking, while only two golfers in the control group did so. Then, MFP was applied with young figure skaters by Bernier et al. [23]. Figure skaters significantly improved their mindfulness and acceptance skills. They obtained a substantial improvement in their competition scores compared to the control group. One of them highlighted the positive effect of MFP on attention regulation in a young skater who practiced all the recommended mindfulness sessions. She became more able to deal with internal and external disturbance and maintain her attention on relevant foci. These preliminary results encourage to confirm the effects of MFP on attention regulation and on real performance in ecological sport contexts. 

Bernier et al. [23] suggested that adherence to the programme (i.e., to follow the recommendation of mindfulness practice) could be a key factor to obtain benefits of MFP. A few studies have found that the positive effects of MABIs on attention or other cognitive skills are a function of personal commitment, in particular, their adherence to practice [33,34,35]. Only one study addressed this issue with athletes: Scott-Hamilton et al. [11] showed that athletes high in adherence obtained significantly greater increases in mindfulness and aspects of flow, and significantly greater decreases in pessimism and anxiety than athletes low in adherence. However, the influence of adherence in MABIs on actual sport performance outcomes was never examined and deserves more attention. The main aim of the present studies was to strengthen the empirical evidence of the efficacy of the MFP programme on real and objective performances in ecological sport contexts. The secondary aim was to examine several processes of change (mindfulness skills development, adoption of foci of attention, adherence to the programme). Based on a group comparison, the first study examined the effects of the MFP programme on mindfulness skills and free-throw performance in ecological training and competition contexts. Using the single-case research design, the second study evaluated the effects of MFP on mindfulness skills, focus of attention and real performance in table tennis, depending on the adherence to the programme.

## 2. Study 1: Effects of MFP on Mindfulness Skills and Free-Throw Performance in Basketball

The aim of this first study was to examine the effects of MFP on mindfulness skills and free-throw performance. An experiment with two groups was set up in a basketball team.

### 2.1. Method

#### 2.1.1. Participants

A total of 17 male Tunisian basketball players aged from 20 to 24 (*Mage* = 22.1, *SD* = 1.4 years), playing at a national level, volunteered to participate in this study. They had been practicing basketball for an average of 14 years. They trained in basketball 2 h a day, 5 times a week for a total of 10 h a week, in addition to 4 h and 30 min strength and conditioning (1 h and 30 min, 3 times per week). Participants affirmed they had never used any formal mental training, as it was not taught in their country.

#### 2.1.2. Design

Participants were randomly divided into two groups: an experimental MFP group composed of 9 participants (*M*age = 22.2, *SD* = 1.4) who followed the MFP programme, and a waitlist group (WL) of 8 participants (*M*age = 22, *SD* = 1.5). The latter group received the MFP programme after the end of the study. Both groups received the same amount of strength and conditioning and basketball training programmes. 

#### 2.1.3. Procedure

All participants received a clear explanation of the study, including the risks and benefits of participation, after which they all agreed to take part in the intervention. The Tunisian research institution in charge of this study requires the involvement of an ethics committee for medical or physiological purposes, but does not monitor the examination of psychological interventions with questionnaires integrated into sport training, as was the case for this study. Data collection and intervention formed part of the team’s training, whereby players were frequently assessed across the season. Therefore, the normal ethics committee clearance was not required.

Before the beginning of the programme, participants of the experimental group were given a presentation of the MFP programme and were told how to complete the questionnaires online; they could ask questions at their leisure. Then, they followed an MFP session that lasted 30 min before each basketball session, five times a week, over six weeks. In each session, explanations were given by the experimenter to foster the understanding of mindfulness and acceptance principles. A mindfulness practice with a 6-to-10-min audio-recording was implemented and combined with a collective debriefing to share and resolve difficulties and to reinforce mindfulness principles. “Homework” was given regarding the autonomous practice to perform in the following week (number of scans to practice alone) and the application of the principles in basketball, such as adopting their personal focus of attention in free-throw and integrating scans at the beginning of the routine and during game pauses. 

The detailed content and adaptation of MFP to basketball are described in Table 1. As highlighted by several authors [8,11,13,23,36], the adaptation of MABIs to the specificities of the sport and context favours the efficacy of the programme. The adaptation was made by the first author together with the last author, who had previous experience in adapting MFP to several sports. The intervention was run by the first author. 

#### 2.1.4. Measures

Mindfulness skills were assessed before the MFP session at T1, after the six-week MFP intervention (T2), and six weeks after the end of the intervention (T3), with the French version of the Mindfulness Inventory for Sport (MIS; [37]) comprising three subscales. First, awareness is the athlete’s ability to recognize disruptive internal experiences (thoughts, emotions, and bodily sensations) that arise in the stream of consciousness and their associated internal reactions (e.g., “I am able to notice the sensations of excitement in my body”). Second, acceptance relates to accepting the presence of disruptive stimuli without judging oneself for experiencing them (e.g., “When I become aware that I am really upset because I am losing, I criticise myself for reacting this way”). Third, refocusing refers to switching the focus of attention from disruptive stimuli to the key elements of performance (e.g., “When I become aware that I am really excited because I am winning, I stay focused on what I have to do”). Responses are made on a 6-point Likert-type scale ranging from 1 (not at all) to 6 (very much). The values of Cronbach’s alpha at T1 were: α_awareness_ = 0.84, α_acceptance_ = 0.86, and α_refocusing_ = 0.90. Participants took the online version of the MIS on mindeval.com website, with individual codes to assure data security.

Free-throw performance was assessed during the training sessions by the experimenter (1 point if the ball went in, 0 if not). Free-throw performance is usually assessed by a series of 10 or 20 tries (e.g., [18]). In the present study, players in both groups performed two consecutive free throws five times every twenty minutes during the 2-h basketball training session. Splitting the free throws into five pairs of two tries was deemed similar to what happens during a game, and thus, more ecologically valid. We recorded the number of points out of the 10 tries. Free-throw performance was measured once a week for 8 weeks (i.e., initial evaluation, the week before the beginning of the intervention, T1; the 6 weeks of the intervention, T2 to T7; and final evaluation, the week after the end of the intervention, T8). In addition, game scoresheets were used to collect free-throw data from the last game before the intervention and from the first game after the intervention. 

#### 2.1.5. Data Analysis

Statistical analyses were carried out using JASP (Version 0.16.2,Amsterdam, Netherlands) [38]. We computed the estimation of the mean and standard deviation. Internal consistency of the scales was appraised using Cronbach alphas. ANOVA repeated over time was conducted to compare the effects of training (groups: MFP vs. WL) on mindfulness skills and on free-throw performance. Post hoc Tukey tests were processed for multiple pairwise comparisons. We calculated the effect size and power for each test. The level of significance used when interpreting and analysing the data was set at 0.01.

### 2.2. Results

#### 2.2.1. Pre-Intervention Comparisons

No significant pre-intervention differences between MFP and WL groups were reported on any dependent variables of the study (see Table 2): awareness, *t* (1, 15) = 1.62, *p* = 0.13, *Cohen’s d* = 0.785; acceptance, *t* (1, 15) = −1.07, *p* = 0.30, *Cohen’s d* = 0.48; refocusing, *t* (1, 15) = 0.39; *p* = 0.34 *Cohen’s d* = −0.521; and free-throw performance, *t* (1, 15) = −0.27, *p* = 0.79, *Cohen’s d* = −0.13. 

#### 2.2.2. Effects of MFP on Mindfulness Skills

Three ANOVAs repeated over time (T1 to T3) were conducted to compare the effect of training (MFP vs. WL) on each of the three subscales of the MIS (See Table 2). ANOVA on awareness revealed a significant effect of group (*F* (1, 15) = 57.51, *p* < 0.001, *η*^2^ = 0.793), of time (*F* (2, 30) = 25.36, *p*< 0.001, *η*^2^ = 0.628) and of group × time interaction (*F* (2, 30) = 27.72, *p* < 0.001, *η*^2^ = 0.649). ANOVA on acceptance revealed a significant effect of group (*F* (1, 15) = 8.22, *p* = 0.012, *η*^2^ = 0.354), of time (*F* (2, 30) = 19.26, *p* < 0.001, *η*^2^ = 0.562) and of group × time interaction (*F* (2, 30) = 53.20, *p* < 0.001, *η*^2^ = 0.780). ANOVA on refocusing revealed a significant effect of group (*F* (1, 15) = 37.23, *p* < 0.001, *η*^2^ = 0.713), of time (*F* (2, 30) = 50.01, *p* < 0.001, *η*^2^ = 0.769), and of group × time interaction (*F* (2, 30) = 14.36, *p* < 0.001, *η*^2^ = 0.489). 

Tukey’s HSD test found that the MFP group improved significantly between T1 and T2 in awareness (*p* < 0.001, 95% C.I. = [−2.34, −1.17], Cohen’s *d* = −9.579), in acceptance (*p* < 0.001, 95% C.I. = [−2.84, −1.59], Cohen’s *d* = −11.27), and refocusing (*p* < 0.001, 95% C.I. = [−4.05, −2.09], Cohen’s *d* = −3.72); and between T1 and T3 in awareness (*p* < 0.001, 95% C.I. = [−2.18, −1.02], Cohen’s *d* = −8.73), acceptance (*p* < 0.001, 95% C.I. = [−2.45, −1.20], Cohen’s *d* = −9.29), and refocusing (*p* < 0.001, 95% C.I. = [−1.76, 0.76], Cohen’s *d* = −0.61). They also showed significant differences between the MFP group and WL group at T2 in awareness (*p* < 0.001, 95% C.I. = [1.48, 3.27], Cohen’s *d* = 8.5), acceptance (*p* < 0.001, 95% C.I. = [0.76, 3.62], Cohen’s *d* = 5.08), and refocusing (*p* < 0.001, 95% C.I. = [1.48, 4.00] Cohen’s *d* = 3.32); and at T3 in awareness (*p* < 0.001, 95% C.I. = [1.41, 3.21], Cohen’s *d* = 8.25), acceptance (*p* < 0.01, 95% C.I. = [0.35, 3.20], Cohen’s *d* = 4.13), and refocusing (*p* < 0.001, 95% C.I. = [1.10, 3.62], Cohen’s *d* = 2.86).

#### 2.2.3. Free-Throw Performance

**Figure 1:**
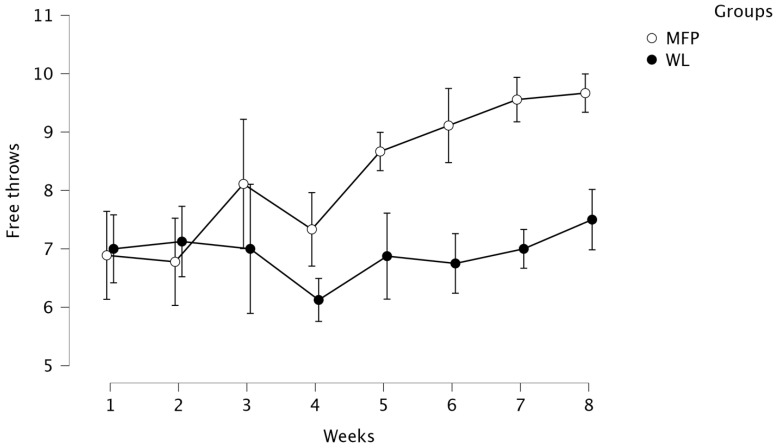
Average of successful free throws during training sessions per week. Note: week 1 = before the intervention, week 1 to 7 = intervention, week 8 = one week after intervention.

A repeated ANOVA over time (T1 to T8) was conducted to compare the effect of training (MFP vs. WL) on free-throw performance (i.e., number of successful free throws, see Figure 1). There was a significant effect of group (*F* (1, 15) = 86.38, *p* < 0.001, *η*^2^ = 0.852), of time (*F* (7, 105) = 11.53, *p* < 0.001, *η*^2^ = 0.435), and of group × time interaction, *F* (7, 105) = 7.65, *p* < 0.001, *η*^2^ = 0.338).

Tukey’s HSD test showed that the free-throw performance of the MFP group improved significantly between T1 and T6 (*p* < 0.001, 95% C.I. = [−3.62, −0.82]), T1 and T7 (*p* < 0.001, 95% C.I. = [−4.07, −1.26]), and T1 and T8 (*p* < 0.001, 95% C.I. = [−4.18, −1.37]). They also found that the free-throw performance was significantly different between MFP and WL at T6 (*p* < 0.001, 95% C.I. = [0.91, 3.81]), T7 (*p* < 0.001, 95% C.I. = [1.11, 4.00]), and T8 (*p* < 0.001, 95% C.I. = [0.72, 3.61]).

Concerning the number of successful free throws during competition games, the analysis of game sheets indicates that athletes from the MFP group recorded a 50% success rate in free throws for the last game before the intervention (17/34), and this score increased to 69% on the first game after the intervention (9/13). In the WL group, the success rate in free throws was 50% at the last game before the intervention (12/24) and 53% at the first game after the intervention (16/30).

## 3. Study 2: Effects of MFP on Mindfulness Skills, Focus of Attention, and Performance in Competition in Table Tennis

The aim of the second study was to assess the effects of MFP on mindfulness skills (in sport and in everyday life), on focus of attention, and on real and global performances in table tennis competitions, depending on the adherence to the programme. A single-case design [39] was chosen for this study because it was relevant to supplement results from group studies by offering flexibility in applied settings and allowing to examine intra-individual variability in key variables and the functional relations between these variables. 

### 3.1. Method

#### 3.1.1. Participants

Ten male table tennis players (*Mage* = 14.14; *SD* = 0.9), ranked nationally, volunteered to participate in the current study. Participants had been practicing table tennis from 4 to 8 years and they trained for at least 10 h per week. They had no previous formal training in psychological skills nor in mindfulness. 

#### 3.1.2. Procedure

The third author presented the project first to the coach, then to the group of athletes. Parents of the participants signed a consent form which explained the purpose, procedures, and advantages of the study as a supplement for table tennis. They were informed that data would remain anonymous, and that withdrawal was possible at any time without needing justification. Application for institutional ethical approval for this study was addressed to Paris Nanterre University ethics and research committee.

While the external validity of a single-case design is lower than in an omographic design, a single-case design provides stronger internal validity—without the need for a control or alternative condition group, as each participant serves as his or her own control [35]. To increase the external validity, we recruited 10 participants and implemented one single-case design per participant. We used an ABA design, with three weeks of data collection during the baseline (phase A), followed by a 6-week MFP intervention (phase B) and a follow-up period without intervention (phase A). In addition, after the beginning of the first participant, we delayed the onset of the intervention byone week for each participant so that any potential effect could not be attributed to maturation or an external simultaneous event in the table tennis training, but rather to the MFP programme itself, as it was processed at a different time for each participant. 

The MFP programme was adapted to table tennis and was conducted by the third author (Table 3). The programme of the week and the various mindfulness exercises with audio files were presented each Monday during the intervention phase. During the week, athletes were invited to practice every day autonomously with audio recordings. Weekly individual meetings took place after training sessions to address the difficulties encountered and the progress made. The coach became familiar with the MFP programme and adapted his discourse to the players. 

The experimenter was present during one 2.5-h MFP session per week for 6 weeks. She led a 6–10 min mindfulness session, individually debriefed the work that was completed in the previous week, and provided homework for each of the remaining days of the week—for example, listening to 6–10 min mindfulness recording. Adherence to the MFP program and personal focus of attention use before serving was recorded.

#### 3.1.3. Measures

The French version of the Five Facets Mindfulness Questionnaire-short form (FFMQ-SF; [40,41]) was administered to participants to assess mindfulness skills in everyday life. FFMQ-SF is a 15-item instrument made up of five dimensions [42]: observing (e.g., “When I’m walking, I deliberately notice the sensations of my body moving”), describing (e.g., “I’m good at finding words to describe my feelings”), acting with awareness (e.g., “When I do things, my mind wanders off and I’m easily distracted”), non-judging of inner experience (e.g., “I tell myself I shouldn’t be feeling the way I’m feeling”), and non-reactivity to inner experience (e.g., “I watch my feelings without getting lost in them”). Responses are made on a 5-point Likert-type scale ranging from 1 (never or very rarely true) to 5 (very often or always true). The values of Cronbach’s alpha were: α_Observing_ = 0.72, α_describing_ = 0.77, α_awareness_ = 0.83, α_non-judging_ = 0.71, and α_non-reacting_ = 0.40.

The French version of MIS [37] was used to assess mindfulness skills in the context of sport. Participants completed online versions of the MIS and FFMQ questionnaires during the baseline phase (phase A, 3 measures), the intervention (phase B, 2 measures), and in the follow-up (phase A, 2 measures) during a period from January to May. The values of Cronbach’s alpha at baseline were: α_awareness_ = 0.70, α_acceptance_ = 0.73, and α_refocusing_ = 0.72. The online versions of the mindfulness tools were administered through mindeval.com website. Two reminders were sent to the participants by email and on their cell phone by text messages.

The performance of each participant was measured in terms of accumulated points and was collected from the published results of table tennis tournaments at each phase.

The percentage of adherence to the individualized focus of attention was collected every week throughout the intervention. Each week and at the end of the training session, we asked all participants to assign a percentage of adherence to the relevant focus of attention chosen at the first step of the programme with the help of the experimenter. 

The adherence to the programme was assessed by asking each participant at the end of the intervention phase to estimate the amount of daily practice completed during the 6 weeks regarding the recommended autonomous practice. It consisted in a percentage with 100%, meaning that the participant had practiced all the recommended daily sessions.

#### 3.1.4. Data Analysis

Analysis for all data variables was performed through a combination of visual inspections and effect-size measurements, which were yielded from the percentage of data exceeding the median. A visual inspection of the graphics was carried out to assess the changes between the baseline, the intervention phase, and the follow-up phase. A visual analysis of the graphed data has been the traditional method for evaluating treatment effects in single-case research [43]. In addition to the visual inspection, the effect size using the percentage of data exceeding the median (PEM) was calculated. PEM is based on the assumption that if the intervention is effective, data will predominately correspond to a positive effect of the baseline median; if an intervention is ineffective, the data points in the intervention phase will vacillate above and below the baseline median [44].

### 3.2. Results 

Out of the 10 participants, 3participants did not respond to the questionnaires (participant 2, 5, and 6) during the intervention phase and were, therefore, excluded from the study. In addition, participants 7, 8, 9, and 10 did not fill the questionnaires during the follow-up phase. 

#### 3.2.1. Adherence to the Programme

The estimated amount of daily practice completed regarding the recommended amount during the 6 weeks varied from 30% to 80% (average of 54.3%): 30% (1 participant); 50% (1 participant); 70% (3 participants); 80% (2 participants) (see Table 4). 

#### 3.2.2. Effects of MFP on Mindfulness Skills

The calculation of PEM of seven participants for FFMQ subscales (see Table 5) revealed a significant change in observation scores with only three participants (participants 1, 7 and 9) following the MFP program. Two participants (participants 4 and 7) revealed a significant change in describing scores following MFP training. Two participants (participants 4 and 7) increased acting with awareness scores following MFP training. However, no significant improvement was recorded for the non-reaction subscale and only participant 4 showed improvement in the non-judging score following the program. 

The calculation of PEM of seven participants for MIS subscales (see Table 6) showed a significant change in awareness score only with participant 9 following the MFP program. Only participant 7 recorded a significant change in acceptance score following the intervention. Four participants (participants 1, 7, 8 and 9) showed a high improvement in refocusing scores following the MFP training. 

#### 3.2.3. Effects of MFP on Focus of Attention

The monitoring of the evolution of the percentage of adherence to a relevant focus of attention for performance during the intervention phase (See Table 7) showed that all participants had improved percentages of adherence to their focus of attention between week 2 and the last week of the intervention phase. Participant 1 recorded the highest percentage and improvement of adherence to the point of attention, which increased from 10% to 100% between the second and the last week of the intervention. Participant 10 recorded the lowest percentage and improvement of adherence to the point of attention, which increased from 5% to 30% between week 2 and the last week of the intervention phase.

#### 3.2.4. Effects of MFP on Performance

All participants (1, 3, 4, 7, 8, and 9) improved their performances in terms of accumulated points during table tennis meetings except participant 10 (See Table 8). The number of points accumulated by participant 10 remains almost stable at the end of intervention phase and registers a slight decrease during the follow-up phase. 

#### 3.2.5. Results of the Cases 1 and 10

**Figure 2:**
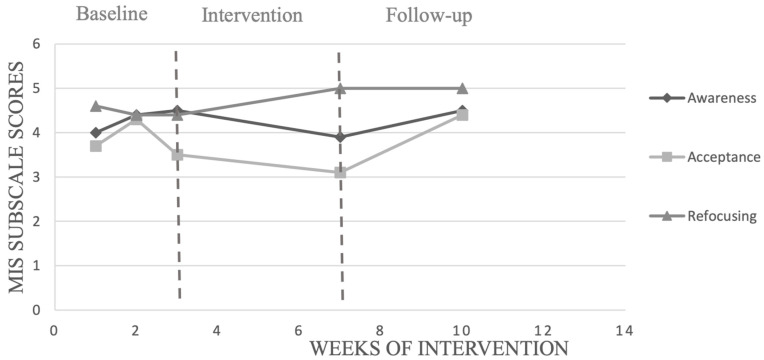
Evolution of scores obtained on the MIS by participant 1, across the weeks of interventions.

**Figure 3:**
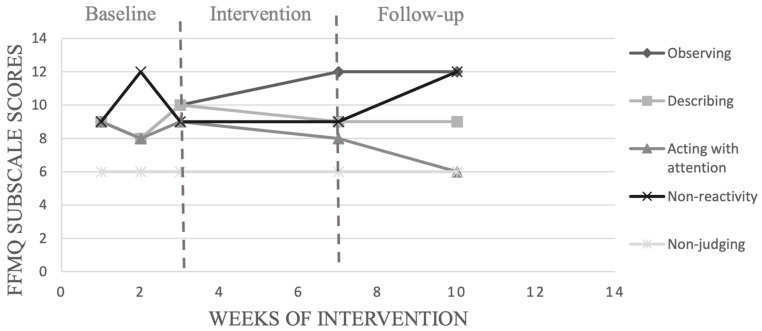
Evolution of scores obtained on the FFMQ with participant 1, across the weeks of interventions.

**Figure 4:**
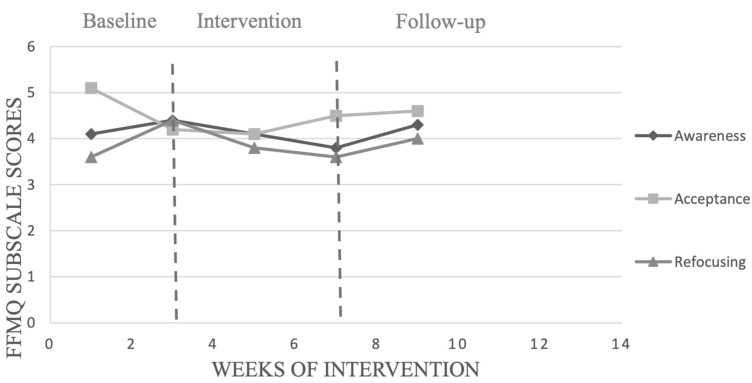
Evolution of scores obtained on the MIS by participant 10, across the weeks of interventions.

**Figure 5:**
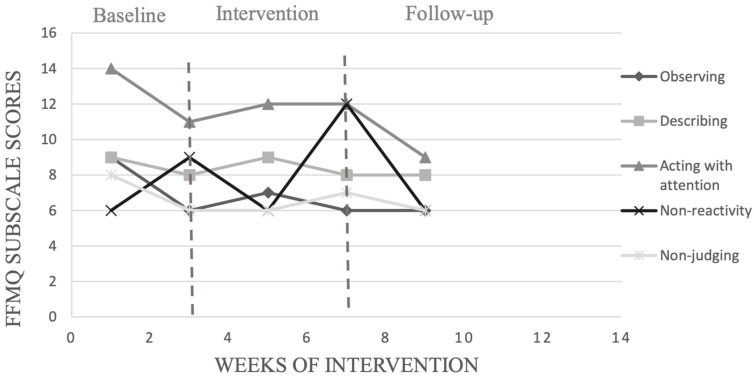
Evolution of scores obtained on the FFMQ by participant 10, across the weeks of interventions.

We chose to present in detail the results of the participants with the highest (participant 1) and the lowest (participant 10) adherence to the training program. Figure 2, Figure 3, Figure 4 and Figure 5 show, respectively, the evolution of the MIS and FFMQ scores across the weeks of the intervention for participants 1 and 10.

Visual inspection of data with participant 1, who recorded the highest percentage of adherence to the programme, revealed an increase in refocusing and observing after the baseline, as shown in Figure 2 and Figure 3. This increase was also visible for acceptance and non-reactivity during the follow-up phase. However, only the increase in refocusing and observing after the baseline and during the follow-up phase was statistically significant with a rate of 100%. Participant 1 also had the highest percentage of adherence to the focus of attention, which increased from 10% in week 2 of the intervention programme to 100% in the last week of intervention (week 6). The number of points scored during competition by participant 1increased from 1824.55 before the intervention to 1860.3 at the end of the intervention. This increase continued during the follow-up phase when the number of points went from 1860.3 to 1881 points, as shown in Table 7. 

For participant 10, who recorded the lowest percentage of adherence to the programme (30%), the visual inspection only showed improvement once with non-reactivity after being exposed to MFP, as shown in Figure 5, but this did not last in the follow-up phase. On the other hand, the level of the other dimensions decreased after the baseline phase. Participant 10 also recorded the lowest percentage of adherence to the focus of attention, which advanced from 5% in week 2 of the intervention to 30% in the last week of intervention (week 6). The number of points accumulated by participant 10 remained stable: 1341 in the phase preceding the intervention and 1450 at the end of the intervention. However, the number of points accumulated slightly decreased in the follow-up phase, from 1450 to 1445.

## 4. Discussion

The main aim of the two complementary studies in basketball and table tennis was to evaluate the effects of the MFP programme on real performance in ecological situations. The secondary aim was to examine some processes of change: changes in mindfulness skills in both studies, adoption of foci of attention, and adherence to the programme in study 2.

Results of the two studies help strengthen the evidence of the efficacy of MFP on actual sport performance. In study 1, the MFP group significantly improved its free-throw performance during practice compared to the WL group. This result differs from the result found in Wolch et al’s study [18], which did not show any significant effect of a brief mindfulness intervention on free-throw performance under experimental pressure conditions. It confirms that MABIs have positive effects on performance in precision tasks [14,15,16,19,20] due to the ecological conditions of the free-throw performance measure (i.e., pairs of free-throws every 20 min during the 2-h basketball training session, as in a standard game). The performance improvement was maintained after the end of the intervention. While more data should have been recorded during games, the improvement in free-throw percentage between the games directly before and after the intervention might be an indication as to how transfer could occur. 

Whereas study 1 provided findings about one specific aspect of actual performance in basketball (i.e., free-throw, which is a closed-precision skill), study 2 focused on overall performance in table tennis. Six participants who followed MFP improved their performance in terms of accumulated points collected from published results compared with the baseline phase. This result supported the rare previous studies showing that MABI may be particularly effective for overall performance enhancement [9,23].

Considering the effects of MFP on mindfulness skills, study 1 showed that mindfulness skills measured with the MIS, which is specific to sport contexts, improved significantly compared to a WL group between the pre- (T1) and post-intervention (T2) measures. These effects were maintained at T3, which was measured 6 weeks after the end of the intervention, demonstrating that they were not only acute effects and that mindfulness skills were actually developed and retained. In study 2, results on mindfulness skills were more disparate. Refocusing appeared to be the skill most influenced by the MFP programme: four out of seven participants improved their refocusing score. The difference between the significant effects of MFP on mindfulness skills in study 1 and the more contrasted effects in study 2 could be explained by two specific hypotheses. The first one concerns the mindfulness practice modalities. In study 1, practice was systematized (five sessions per week) and guided by an experimenter, while in study 2, only one weekly session was conducted by an experimenter and recommendations were given about the sessions that athletes should practice autonomously with audios. Systematized and supervised practice seems to favour the development of mindfulness skills. The explanatory hypothesis may be related to the age of the participants. Table tennis players in study 2 were adolescents, while basketball players in study 1 were adults. The MFP programme may be more efficient to develop mindfulness skills in adults than in young athletes. Previously highlighted by Bernier et al. [8,23], the adaptation of MFP for young athletes seems to be crucial and needs more research. 

Comparing the results of MIS and FFMQ in study 2, inconsistencies could be noted. The results of MIS for awareness and acceptance scores were not consistent with FFMQ subscales results. For example, participant 7 did not register a significant increase in awareness score measured by the MIS but did register a significant increase in acting with awareness score measured by FFMQ. It is likely that mindfulness skills in sport contexts differ from mindfulness skills in everyday life. Moreover, the inconsistencies between MIS and FFMQ results suggest that it is necessary to re-question the measuring accuracy of mindfulness skills in a sport context. Inconsistent results were previously noted by several studies using the MIS [13,36,45]. Improving the existing tools or developing new tools seem needed to better measure the effects of MABIs on mindfulness skills that are specific to sport contexts, particularly in young athletes.

Mindfulness skill development and actual performance seem to be linked. In study 1, effects of MFP on mindfulness skills and on free-throw performance followed the same positive evolution. In study 2, the analysis of case 1 showed improvements both in some mindfulness skills (observing, acceptance, non-reactivity, refocusing) and in actual performance. However, the designs of the two studies do not allow to confirm a causal link between the development of mindfulness skills and performance improvement. The dynamic of this relation throughout the MFP programme should be examined in future research. 

In study 2, the effect of MFP on focus of attention was assessed to verify that theMFP programme is effective in fostering attention regulation in highly demanding situations, as designed [27]. Throughout the intervention phase, all the participants improved their adherence to relevant foci of attention, which were individually defined and adjusted. These findings support previous studies showing that MABIs may improve attention regulation [8,9,13,23]. They confirm that attention regulation could be considered as a process of change involved in the efficacy of MABIs.

Interestingly, the analysis of individual cases in study 2—taking consideration of the adherence to the programme in terms of amount of autonomous practice—revealed that participants who showed the highest percentage of adherence to the programme benefited more from MFP training than participants who showed a weaker percentage of adherence to the programme. This result supplements those of Scott-Hamilton et al.’s study [11], highlighting the influence of adherence to the programme not only on changes in psychological variables but also on performance outcomes. Future interventions with MFP, especially in young athletes, should insist on the means to increase adherence, as it seems that it is a key issue for the success of the programme to enhance performance. Adapting and integrating behaviour change techniques could favour the systematization of autonomous practice (e.g., goal setting, action planning, monitoring of practice, social support, vicarious experiences). The issue of adherence was not raised in study 1 as the sessions were conducted five times per week by an experimenter. Supervised sessions may have ensured sufficient practice and boosted the effects on key variables and performance. We can conclude that when possible, supervised sessions should be emphasized rather than autonomous practice.

## 5. Conclusions

The two present studies deepen knowledge about the efficacy of MABIs and, in particular, the MFP programme in the context of sport and some processes of change (mindfulness skills development, adoption of foci of attention and adherence). In keeping with the first studies, which tested the effects of initial versions of MFP [8,23,27], this research highlights original findings and opens new applied perspectives regarding supervised vs. autonomous practice and the adaptation of MFP to the age of the athletes. The main strength of the two studies relates to the high ecologically valid conditions of intervention and of assessment. They offered information that may guide professional practice. The downsides of the high naturalistic characteristic of the studies (intervention and assessment integrated in real training and competition conditions with athletes from a national level) regard some limitations which are considered in the following section, deeming it necessary to support the trends that emerge in further interventional research.

## 6. Limitations and Future Research

First, generalisation is limited by the small size of the populations. Then, an active control group might have better supported the reliability of the results. In study 2, missing data due to the fact that some participants did not complete all the questionnaires during the intervention or the follow-up phases pose a problem. The young table tennis players did not complete the online assessment as conscientiously as older basketball players did. Missing data could also be explained by the study designs, which involved seven assessment times with two questionnaires in study 2, compared to three assessment times with one questionnaire in study 1. Even if a single-case design is a relevant design to support internal validity, it could be difficult to obtain a suitable rate of response to the repeated-measures design by questionnaires, in particular with young athletes. It might have been more appropriate to implement the measures in the presence of an experimenter rather than completing them online. 

While the MFP programme targets a single goal (i.e., to perform in highly demanding situations by regulating attention on relevant foci), it is a multi-component programme with various contents that are assumed to contribute to this goal at each of the three stages. The designs of the two studies do not make it possible to demonstrate which content determines performance improvement. However, some results of study 1 highlight preliminary findings. It is interesting to note that the effect on free-throw performance was significant from the fifth week of the intervention, while work on attentional foci was carried out in stage 1, and mindfulness and acceptance were introduced and practiced in stage 2. Future research could refine the demonstration of the separate and cumulative effects of the different stages and components of the programme with longitudinal designs involving frequent assessments. This could lead to adjusting and optimizing the content or the structure of MFP.

As Halperin et al. [46] stressed the importance of replicating the studies with the goal to validate the results and inspect their reliability, it could be of great interest to replicate the MFP programme to confirm the present trends and to measure its effects in other disciplines. It is necessary to supplement the assessment of MFP effects on overall performance in different kinds of sports (e.g., dual sports, team sports, endurance sports). Effects on specific indicators of performance could also be evaluated (tactical indicators or technical execution assessed by experts, and physiological and biomechanical indicators).

Future research could allow to better adapt the MFP programme to the context, the population (e.g., adolescents vs. adults), and the specific demands of the sport. This programme is not fixed once and for all [8,23,27]. Practitioners that are willing to apply this need more knowledge to better adjust it to specific settings.

## Figures and Tables

**Table 1:** Adapted content of the six-week MFP programme in basketball.

Weeks	Specific Content for Basketball
1	Definition of an individualized relevant focus of attention for free throw (for example, the middle of the inner-back side of the rim) to be used before each free-throw shot. Introduction to scans (i.e., ask and answer in your mind the two questions ‘What I am thinking about? What do I feel?’) and to the mindfulness sessions which were presented as focusing on breathing (10 min session in a sitting position to notice distractions and refocus on breathing).
2	Remind to use focus of attention before each free throw. Mindfulness practice: ‘focusing on breathing while sitting’ sessions. Remind to practice scans everyday.
3	Remind to use focus of attention before each free throw. Mindfulness practice: ‘focusing on breathing while standing’ sessions. Remind to practice scans and integrate it during basketball session before free throws.
4	Introduction to acceptance (notice and accept thoughts and feelings as transitory mental events). Presentation of sessions with train, cinema, and clouds metaphors (watch the train of thought/feelings, watch a movie of your mental events, watch clouds carrying your mental events). Emphasis is placed on being an observer and on considering mental events as only mental events. Mindfulness practice: ‘focusing on breathing’ sessions and acceptance sessions based on the metaphors. Request to shorten scan to 2 min and increase the number of scans at the beginning of the routine.
5	Mindfulness practice: Acceptance sessions based on metaphors and integrated mindfulness sessions, alternating focus on breathing for 2 min and playing basketball for 2 min, repeated 5 times. Request to increase the number of brief scans at the beginning of the routine.
6	Mindfulness practice: Acceptance sessions based on metaphors and integrated mindfulness sessions, alternating focus on breathing for 1 min and playing basketball for 1 min, repeated 5 times. Request to increase the number of brief scans at the beginning of the routine.

**Table 2:** Average and standard deviation of MIS scores before (T1), after the 6-weekintervention (T2), and 6 weeks later (T3) for the experimental (MFP) and wait list (WL) groups.

MIS		Awareness			Acceptance			Refocusing		
		T1	T2	T3	T1	T2	T3	T1	T2	T3
MFP	Mean	3.4	5.1	5	2.9	5.1	4.8	2.8	5.8	5.6
	SD	0.8	0.2	0.2	0.8	0.2	0.4	1.1	0.3	0.3
WL	Mean	2.8	2.8	2.7	3.5	3	3	2.3	3.3	3.3
	SD	0.6	0.5	0.5	1.2	1.1	1	0.9	0.8	0.9

**Table 3:** Adapted content of the six-week MFP programme in table tennis.

Weeks	Specific Content for Table Tennis
1	Definition of an individualized relevant focus of attention for each player to be used before each service, such as visualizing ball trajectory or focusing on the target. Introduction to scans (i.e., ask and answer in your mind the two questions ‘What I am thinking about? What do I feel?’) and to mindfulness sessions presented as focusing on breathing (10 min session in a sitting position to notice distractions and refocus on breathing).
2	Remind to use focus of attention in the routine before serving. Mindfulness practice: ‘focusing on breathing while sitting’ sessions. Homework: Practice scans everyday.
3	Remind to use focus of attention in the routine before serving. Mindfulness practice: ‘focusing on breathing while standing’ sessions. Homework: Practice scan and integrate it during table tennis session in the routine before serving or receiving.
4	Introduction to acceptance (notice and accept thoughts and feeling as transitory mental events). Presentation of sessions with train, cinema, and clouds metaphors (watch the train of thought/feelings, watch a movie of your mental events, watch clouds carrying your mental events). Emphasis is placed on being an observer and on considering mental events as only mental events. Mindfulness practice: ‘focusing on breathing’ sessions and acceptance sessions based on the metaphors. Homework: Shorten scan to 2 min and increase scan in the routine before serving or receiving.
5	Mindfulness practice: Acceptance sessions based on metaphors and integrated mindfulness sessions, alternating focus on breathing for 2 min and playing table tennis for 2 min, repeated 5 times. Homework: Increase the number of brief scans in the routine before serving or receiving.
6	Mindfulness practice: Acceptance sessions based on metaphors and integrated mindfulness sessions, alternating focus on breathing for 1 min and playing table tennis for 1 min, repeated 5 times. Homework: Increase the number of brief scans in the routine before serving or receiving.

**Table 4:** Adherence to the programme in terms of percentage of practice regarding the recommended daily exercises.

Participants	Percentage of Adherence
P1	80%
P3	80%
P4	70%
P7	70%
P8	70%
P9	50%
P10	30%

**Table 5:** Percentages of data exceeding the median recorded in FFMQ subscales.

Participants	Observing	Describing	Acting with Awareness	Non-Reaction	Non-Judging
P1	100%	0%	0%	50%	0%
P3	25%	25%	75%	0%	25%
P4	0%	100%	25%	0%	100%
P7	100%	100%	66%	0%	0%
P8	33%	0%	0%	0%	0%
P9	100%	33%	33%	0%	0%
P10	0%	0%	0%	33%	0%
Average	51.14%	36.86%	28.43%	11.86%	17.86%

**Table 6:** Percentage of data exceeding the median recorded in MIS subscales from baseline to intervention phase for the seven participants.

Participants	Awareness	Acceptance	Refocusing
P1	0%	50%	100%
P3	25%	50%	25%
P4	25%	50%	50%
P7	0%	60%	100%
P8	33,3%	30%	100%
P9	100%	0%	66%
P10	0%	0%	33%
Average	26.19%	34.29%	67.71%

**Table 7:** Percentages of adherence to focus of attention from week 2 to week 6 of the intervention phase for the seven participants.

Week 2	Week 3	Week 4	Week 5	Week 6
10%	30%	50%	70%	100%
5%	15%	30%	40%	50%
8%	12%	25%	40%	60%
10%	20%	40%	60%	80%
10%	20%	30%	50%	80%
5%	10%	20%	40%	50%
5%	10%	15%	20%	30%

**Table 8:** Performance in terms of points accumulated in competition before, at the end, and after the intervention for the participants.

Participants	Before Intervention	End of Intervention	Follow-Up Phase
P1	1824	1860	1881
P3	1314	1383	1396
P4	1638	1666	1681
P7	1271	1306	1320
P8	1666	1700	1730
P9	935	940	960
P10	1341	1350	1345

## Data Availability

Not applicable.
